# Computerized Axiographic Findings in a Cohort of Migraine Patients: A Cross-Sectional Study

**DOI:** 10.3390/dj12070204

**Published:** 2024-06-30

**Authors:** Nikolaos Zokaris, Marcus Greven, Michail Tzakis, Vasileios Psarras

**Affiliations:** 1 Department of Prosthodontics, 251 Hellenic Air Force and VA Hospital, 15561 Athens, Greece; 2 Department of Prosthodontics, University Clinic of Dentistry, Medical University of Vienna, 1090 Vienna, Austria; markus.greven@meduniwien.ac.at; 3 Department of Orofacial Pain, School of Dentistry, National and Kapodistrian University of Athens, 11527 Athens, Greece; mtzakis@dent.uoa.gr (M.T.); vpsarras@dent.uoa.gr (V.P.)

**Keywords:** migraine, condylography, jaw tracking, computerized axiography

## Abstract

Background: The objective of this work was to investigate the association between the function of the stomatognathic system and migraine presence through an instrumental functional analysis in a group of diagnosed migraine patients and a control group. Methods: This study included 50 individuals in each group. A jaw-tracking analysis was performed using Cadiax 4. Tracings of the following movements were recorded: open/close, protrusion/retrusion, mediotrusion, speech, bruxing, and mastication. The tracings were evaluated for their quantity, quality, transversal characteristics, speed, curvature pattern, and condylar stability. Results: Statistically significant differences between the groups were established for several aspects of the evaluation. Migraineurs presented with (a) higher values of mandibular lateral translation in protrusion/retrusion (*p* = 0.001), open/close (*p* = 0.031), and mastication (*p* = 0.016); (b) transient velocity losses in open/close (*p* = 0.001) and protrusive movements (*p* = 0.018); (c) a compromised condylar stability for protrusion/retrusion (*p* = 0.001) and mediotrusion (*p* = 0.003); (d) a compromised quality for protrusion/retrusion (*p* < 0.001) and mediotrusion (*p* = 0.003); and (e) a more frequent “figure-eight” curvature in open/close (*p* = 0.012). Conclusions: The importance of the stomatognathic function in migraine pathogenesis and treatment should be considered by using a patient-centered and interdisciplinary approach.

## 1. Introduction

Migraines represent a highly incapacitating condition that is prevalent on a global scale. Approximately 15% of the population experiences migraines, making it the second most common type of headache and the second most common among the world’s causes of disability [1]. Migraines are classified as a neurovascular response to both external and internal stimuli that affects the trigeminovascular system [2]. In recent decades, the correlation between the neurological system and the stomatognathic system has grown more apparent [3]. Due to the afferent signal carried into the central nervous system (CNS) via the trigeminal network system, noxious stimuli from the stomatognathic system may initiate a cascade of events [4]. Migraines are linked to anxiety, depression, sleep disturbances, and chronic pain issues, such as neck and lower back pain [5]—regions that are distant from the trigeminal system. Hence, it is crucial to effectively regulate the potential impact of areas that are part of the same system. The existing evidence suggests a higher occurrence of migraines in temporomandibular-disorder (TMD) cohorts, as well as a higher occurrence of TMDs in migraine cohorts [6,7,8,9,10,11,12,13].

The temporomandibular joint (TMJ) is highly complex and crucial in the human body, playing a key role in functions as important as chewing, swallowing, breathing, and speaking. The anatomy and neurophysiology of the TMJ provide the proper foundation and substrate so that it can perform all the aforementioned functions accurately and with the least possible loss of energy. These movements involve a complicated sequence of interconnected three-dimensional rotational and translational activities. 

Condylography instruments are verified to ensure the precise recording of the trajectory of the condyles and the articular disc as they move along the incline of the articular eminence (condylar guidance). The measuring device also records the sagittal condylar inclination (SCI), which is influenced by various factors, such as the geometry of the mandibular fossa, the disc, the related ligaments, the neuromuscular system, teeth morphology, and the articular eminence. The accurate identification of the mandibular hinge axis and a method to track its movement in space and time throughout the functioning of the stomatognathic system are necessary for both diagnostic and subsequent therapeutic purposes. An instrumental functional analysis is a standardized process that aims to record and diagnostically evaluate the mandibular kinematics and function. Jaw-tracking records have been utilized within the dental field for over a century. Since the early 1900s, the concept of localizing and documenting the movement of the mandibular hinge axis has persisted, starting with the research of Gysi [14], Bennet [15], and Campion [16] and continuing into the present digital age. The captured motion patterns offer valuable insights into the morphological state of the joints and can also function as a monitoring tool alongside treatment [17]. The utilization of electronic functional diagnostics has not been widely embraced by the dentistry field. Several researchers have challenged the value of jaw tracking, as evidenced by multiple studies [18,19]. The reasons for this phenomenon could potentially be attributed to several factors. Firstly, the laborious nature of the application could discourage its use. Secondly, there is possibly an established consensus that such advanced condylar recordings are unnecessary for the establishment of a diagnosis and that a clinical examination by itself is sufficient. Thirdly, there is concern that the inappropriate use of the technique may lead to extensive occlusal rehabilitation for the patient [20]. Lastly, cost may also be an issue. The use of computerized axiography, however, is regaining popularity, particularly because functional data can now be imported and incorporated into a virtual patient profile [20]. 

The aim of the present study was to examine the potential disparities in mandibular and condylar kinematics between individuals who experience migraines and a non-headache control group, using computerized axiography. If there was a difference in the recorded movement patterns, suggesting a vulnerability to stomatognathic dysfunction in individuals who experience migraines, it is likely that a multidisciplinary approach, including an oral health professional, could be beneficial for the migraine sufferer. Moreover, in the absence of a clearly established correlation, these findings can still be advantageous, as they can deter individuals suffering from headaches from pursuing unneeded dental interventions in the hopes of alleviating their symptoms.

Thus, the null hypothesis formed was that the results of a motion analysis of the mandible will not differ between migraineurs and people who do not suffer from headaches.

## 2. Materials and Methods

Sample formation: This study was approved by the Ethics and Research Committee of the 251 Hellenic Air Force and VA Hospital (251 GNA/Number: Φ.076/AΔ1348/Σ.48310.02.20/251ΓΝA) and was conducted in accordance with the Helsinki Declaration. Informed consent was obtained from all the participants. We used the online software https://clincalc.com/stats/samplesize.aspx (accessed on 17 November 2019) to determine the proposed sample size, which ranged from 17 to 44 subjects depending on the chosen variable. As a result, we selected 50 subjects per group. Fifty subjects diagnosed with migraines by a neurology specialist in the outpatient headache clinic of the hospital, according to ICHD-3 [21], were asked to enroll in the study and formed the migraine group (MG). Fifty volunteers matched by age (+/− 5 years) and gender were also enrolled, forming the control group (CG). We set the power of the study at 80% and the level of significance at 95% to calculate the sample size. 

All aspects of this study were performed in the prosthodontic department of the hospital. All the participants completed the migraine disability assessment questionnaire (MIDAS). We excluded subjects in the control group with a MIDAS score greater than 1 from the study. The other exclusion criteria for the CG were as follows: (a) active orthodontic treatment; (b) the wearing of a removable prosthesis; (c) missing occlusal contacts until the first molar; (d) no basic English language skills; and (e) an age under 18 years. The same exclusion criteria were applied to the participants in the MG, except for the missing occlusal contacts until the first molar. 

Computerized axiography: Computerized axiography was performed using Cadiax 4 and the corresponding software, the Gamma Dental version 44.6.0.222 document browser (Gamma Dental, Klosterneuburg, Austria). A para-occlusal clutch was used, customized with Pro-Temp 4 (3M ESPE, St. Paul, MN, USA) to fit in the labial and buccal surfaces of the mandibular dentition. The clutch was checked intraorally, and any excess material that created interference in the maximum intercuspal position (ICP) was trimmed away. The para-occlusal clutch was stabilized in the mandibular dentition using the glass ionomer cement Rely-X Luting (3M ESPE, St. Paul, MN, USA).

**Figure 1:**
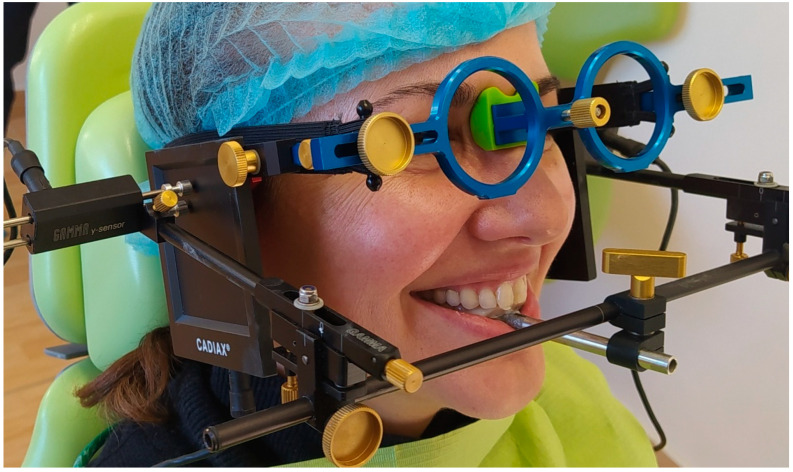
Cadiax 4 assembled on a study participant. The upper and lower facebows, the side arms, the double-stylus system, and the flags are all clearly depicted.

With the clutch in place, the upper and lower facebows, the flags, and the double styli were attached to the patient according to the manufacturer’s instructions. The mandibular bow transmitted the hinge axis movement of the mandible to the upper facebow. The upper facebow carried sagittally mounted flags for the electronic registration of the hinge axis movement. The double-stylus system ensured the accurate determination of the hinge axis position and also allowed for an exact evaluation of the rotational capacities (Figure 1). As a result, it was possible to record functional movements such as bruxing, chewing, and swallowing and analyze them for diagnostic purposes.

With the complex assembled, the dynamic localization of the mandibular hinge axis followed. A rotational opening movement of at least 10 mm was necessary to locate the hinge axis. The stylus, when not in the hinge axis position, made an arc that the computer used to calculate the center of a circle. This center was the hinge axis [22]. The positions of the hinge axis and the stylus were both displayed on the computer screen, and by adjusting the side arms of the lower bow, the stylus point could be moved until it coincided with the hinge axis point. Hence, to be localized, the hinge axis needed to be registered in the recording system.

Every patient executed the following movements in order for their tracings to be recorded: (a) protrusion/retrusion (P/R); (b) right mediotrusion; (c) left mediotrusion; and (d) open/close (O/C) movements. To ensure reproducibility, the patients repeated each movement three times. We also recorded functional movements, which included (a) speech, by asking the patient to count out loud from seventy to sixty, (b) bruxing, and (c) masticating a provided piece of chewing gum. The starting position was the maximum intercuspal position (ICP), which was chosen in order to start as close to occlusion as possible, thus enabling the influence of occlusion on the executed movements to be investigated. The operator refrained from directing any of the movements.

**Figure 2:**
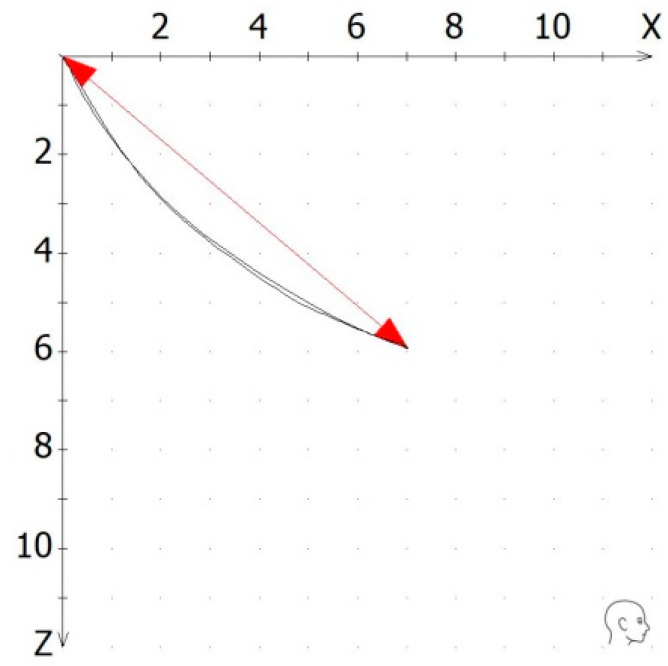
The quantity of a recorded tracing was calculated as the spatial distance between the starting position and the most excursive point in the x/z coordinate system.

**Figure 3:**
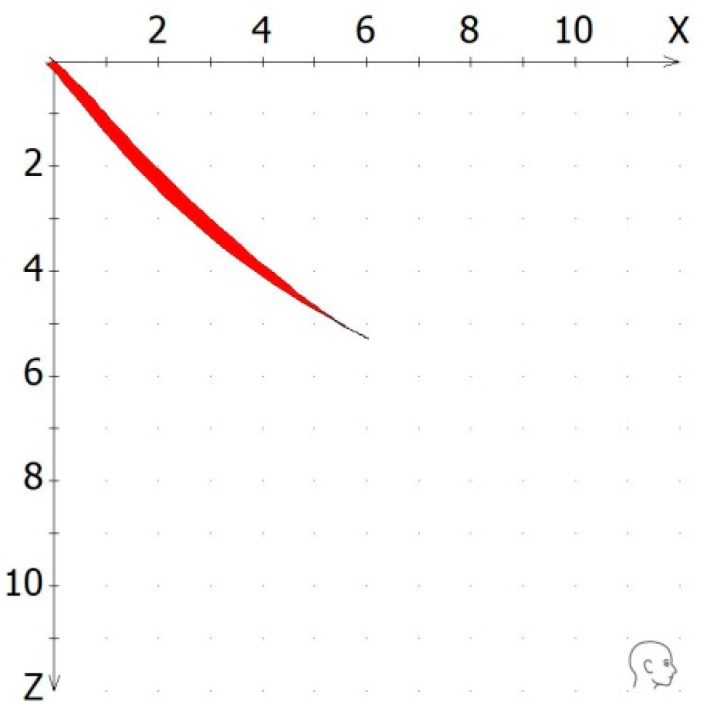
The quality of a recorded tracing was calculated as the ratio of the tracing’s inner area and an excursion distance of 5 mm or 10 mm in the x/z coordinate system.

**Figure 4:**
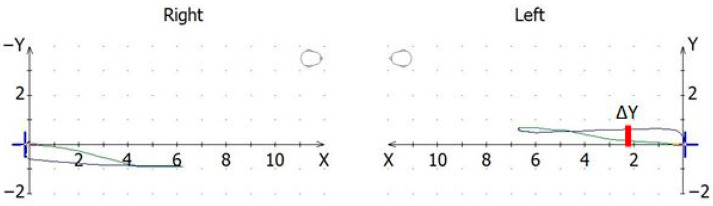
The ΔY of a recorded tracing was calculated as the distance of the tracing from the x axis to the point of maximum medial deviation in the x/y coordinate system.

**Figure 5:**
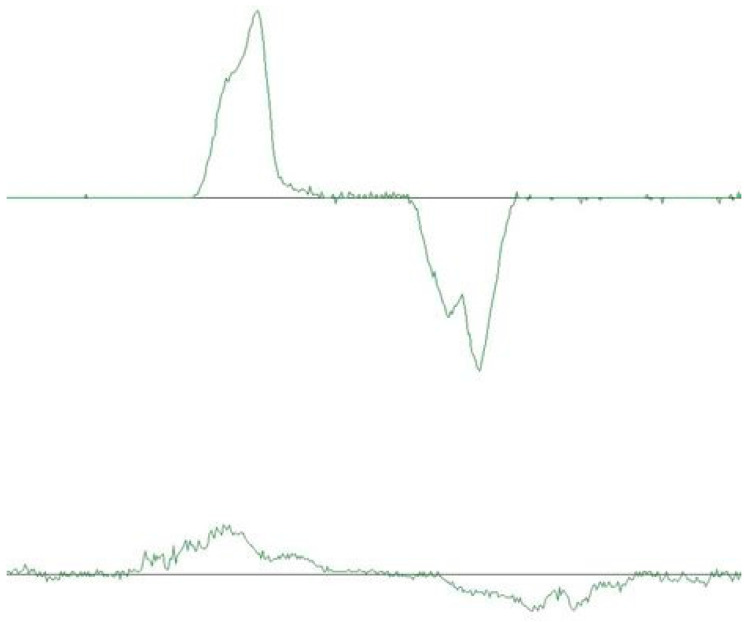
Two different velocity profiles are presented in this figure. The upper represents a one-peak velocity curve while the lower represents a multi-peak velocity profile.

**Figure 6:**
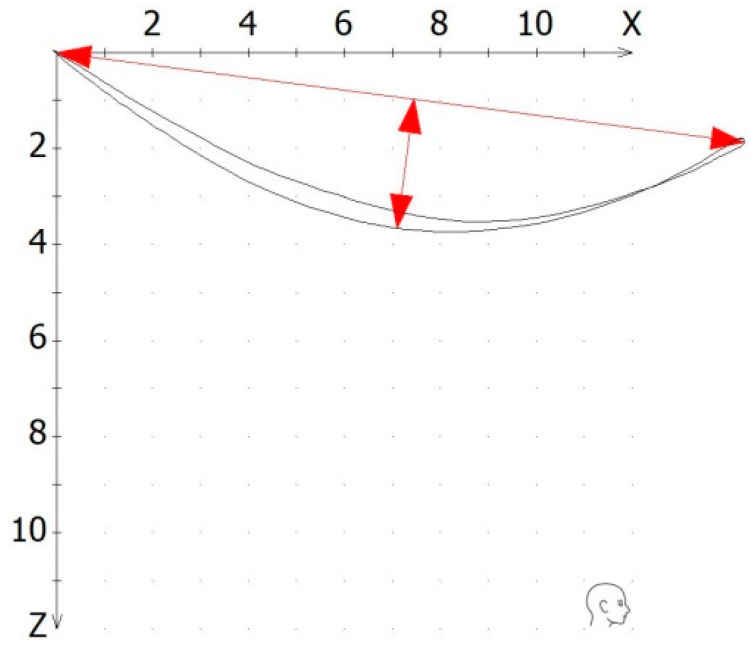
The straightness of the recorded tracings was calculated as the relationship of the maximum excursion distance to the farthest distance between the curve and a straight line.

**Figure 7:**
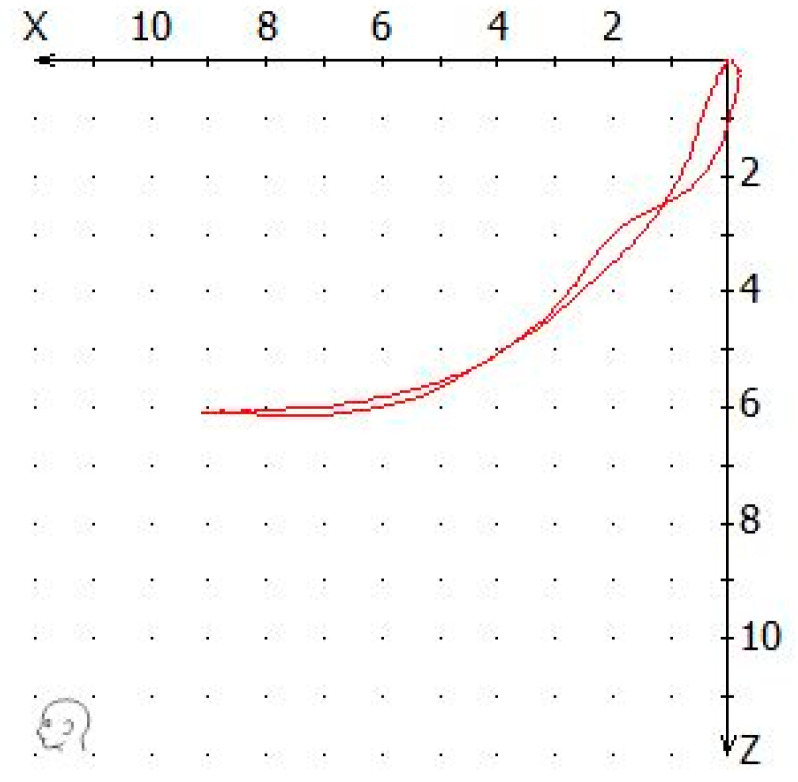
If the incursive and excursive tracings intercrossed, a “figure eight” was depicted.

**Figure 8:**
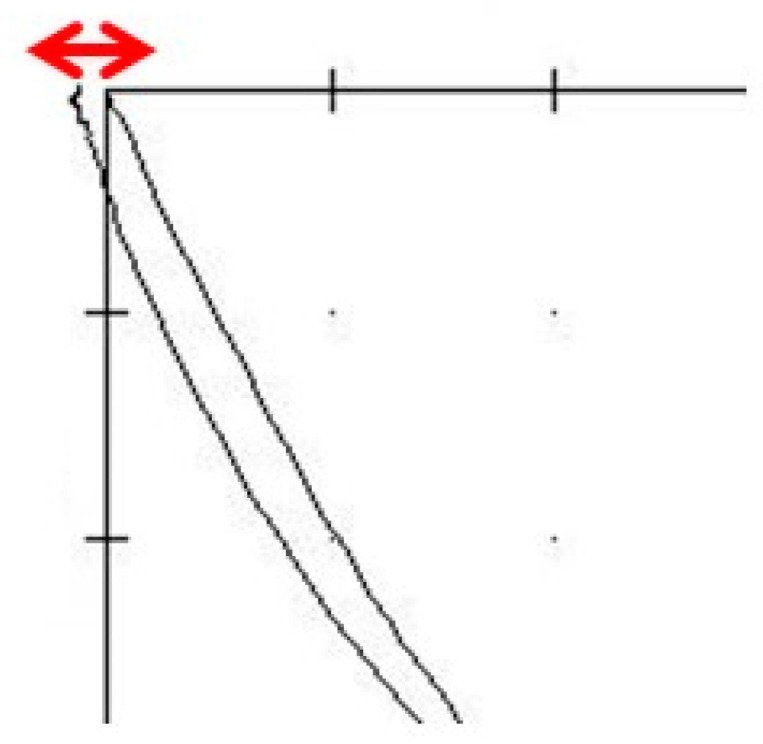
The condylar stability of the tracing was calculated as the distance between the start point and the end point of the tracing.

Variables studied: In order to ensure objective data, only the numerical values were derived for the analysis. The software allowed for a numerical analysis of all the tracings. The dependent variables of the study were as follows:

(1) Quantity: The quantity of each movement was recorded. This value was calculated as the spatial distance between the starting position and the most excursive point of the tracing. It was represented as the “S” secant from the starting position to the end point of the excursion (Figure 2).

(2) Quality: The quality was generally assessed by the operator as excellent, average, or poor. Tracings of an excellent quality were presented as an exact line with no or minimal deviation between the excursive and incursive parts of the tracing. However, since we decided to use only objective numerical data, the numerical value of “reproducibility” was utilized to evaluate the quality. This value was calculated as the ratio of the tracing’s inner area and an excursion distance of 3 mm, 5 mm, or 10 mm. Thus, by maintaining the same denominator, higher values of reproducibility expressed a greater inner area of the tracing, which in turn described a greater deviation between the excursive and the incursive part of the tracing, and thus, a lower quality (Figure 3).

(3) Mandibular lateral translation in symmetrical and functional movements (ΔΥ): This value was always calculated for the side with the medial deviation (Figure 4).

(4) Speed phenomena: A normal velocity profile is characterized by a largely one-peak velocity curve during jaw-opening and jaw-closing movements. However, the condylar velocity may exhibit a two-peak or multi-peak profile, indicating transient velocity losses (Figure 5).

(5) Curvature pattern/Kobs coefficient: This value describes the straightness of the tracing and is calculated as the relationship of the maximum excursion distance to the farthest distance between the curve and a straight line. Values of less than 0.05 indicate a predominantly straight tracing and higher values indicate a curved tracing. Straight tracings are suggestive of intra-articular disorders, while concave tracings are indicative of a healthy joint (Figure 6).

(6) “Figure-eight” tracing: When the incursive and excursive tracings intercrossed, a “figure-eight” tracing was depicted, which is indicative of intra-articular disorders (Figure 7).

(7) Condylar stability: This value was calculated as the distance from the start point to the end point of the tracing (Figure 8).

### Statistical Analysis

The qualitative variables are reported as numbers, while the quantitative variables are expressed as the mean and standard deviation. To determine whether the qualitative variables were independent of the group variable, we used the chi-square test. To determine whether the quantitative variables were independent of the group variable, we used a *t*-test or the Mann–Whitney test. The statistical significance was set at 5%. All the analyses were conducted using SPSS version 22.0.

## 3. Results

A total of 50 subjects were recruited in each group. We matched the two groups by age (+/− 5 years) and gender, with each group consisting of 37 females and 13 males. The mean age of the sample was 39 years (40.7, MG–37.3, CG). The MIDAS score for the MG was 29.48, their pain intensity was 6.7 on a 0–10 scale, their average number of headache days per month was eight, and 72% of the patients were categorized in grades 3 and 4, suggesting a medium to severe disability. A statistically significant difference could be established for the following:

- The mandibular lateral translation (ΔΥ), which was significantly higher in subjects who experienced migraines during protrusion (*p* = 0.001), open/close movements (*p* = 0.031), and mastication (*p* = 0.016).
- The velocity, which seemed to appear with transient losses in the open/close (*p* = 0.001) and protrusive movements (*p* = 0.018) in migraineurs.
- The condylar stability, which was significantly compromised in the migraine group for protrusion (*p* = 0.001) and mediotrusion (*p* = 0.003).
- The quality, which was significantly compromised in the migraine group for protrusion (*p* < 0.001) and mediotrusion (*p* = 0.003).
- A “figure-eight” curvature, which was found more often in the migraine group (*p* = 0.012).

All the data are presented in the following tables (Table 1, Table 2, Table 3, Table 4, Table 5 and Table 6). 

## 4. Discussion

Computerized axiography is a valuable, non-invasive method that can be used to record mandibular motion in space and time. It is important to note that axiography is just one component of the diagnostic cascade. A comprehensive medical and dental record and a detailed clinical examination are equally important. However, axiography offers a comprehensive and detailed overview of mandibular kinematics. The data retrieved are actually a quantification of orthopedic diagnostics with a high level of accuracy [22]. Jaw tracking is an accurate evaluation method for detecting internal derangements in the joint, with a sensitivity of 0.7 and a specificity of 0.8 [23]. Compared to classical MRI, it records the dynamic function of the structures, but also allows for an estimation of the degree of successful adaptation to a current disorder [24]. A detailed analysis of the tracings offered the possibility of detecting abnormal or pathological situations, such as muscular dyscoordination, hyper-/hypomobility, dynamic asymmetries of movement, avoidance mechanisms, and joint pathologies, even at an early stage, which could otherwise remain undiagnosed; therefore, this can improve the accuracy of clinical diagnoses [25]. Moreover, the recording of movements can reveal avoidance mechanisms and/or asymmetries during function [22]. Jaw-tracking tracings are interpreted in terms of their quantity, quality, symmetry, curvature characteristics, reproducibility, transversal characteristics, and condylar stability, and this pattern was used to define the dependent variables of our study. In 2013, the German Society for Functional Dentistry published a paper with guidelines regarding the importance and interpretation of condylographic findings [26]. This paper was revised one year later [17]. Our investigation adhered to the fundamental concepts outlined in these guidelines.

The quantity is a record of the maximum movement of the hinge axis, and depending on the value derived, it can be categorized as hypomobile, normal, or hypermobile. The extent of hinge axis movement is determined by morphology and function [27]. A range of 10–14 mm for protrusion and mediotrusion is considered normal. The stylomandibular ligament acts as the physical limit of the protrusive tracing; however, values of less than 10 mm are considered suspicious and hypomobile [28]. For open/close tracings, the normal range varies from 12 to 16 mm. In a group of 186 healthy volunteers aged between 18 and 21 years, Kondrat et al. [29] evaluated the quantity using Cadiax Compact 2. They reported a max “S” for open/close movements of 13.3 mm, which is very close to our finding of 13.94 mm for the control group. Piehslinger et al. [30] evaluated the quantity of protrusion in 225 individuals—45 volunteers and 180 TMD patients—using computerized axiography. Among the healthy volunteers, the mean length of protrusion was 9.77 mm and 10.91 mm, depending on gender. Among the TMD patients, the mean lengths of protrusion were 9.01 mm and 9.65 mm. The greater lengths corresponded to males. Kawagoe et al. [31] examined 240 participants, of whom 72.6% were free of jaw pain. The average quantity for open/close was 13.5 mm; for protrusion, it was 8.7 mm; and for mediotrusion, it was 9.5 mm.

In our study, the only statistically significant difference established regarding quantity was for the length of the protrusion/retrusion record, and only when all the joints were incorporated and the sides were unified. There was no difference between the right and left sides when examined separately. The MG presented a mean length of 9.43 mm, just below what is considered normal. The possible reasons for hypomobility include muscle pain, muscle hardening, disc–condyle shifts, arthritis, ankylosis, luxation or subluxation, a dry joint, or the contracture of the capsule [27,30,32,33,34].

The quality represents homogeneity, and ideally, tracings of an excellent quality are presented as an exact line with no or minimal deviation between the excursive and incursive parts of the tracing. In general, the quality is assessed by the clinician as excellent, moderate, or poor. However, in our study, in order to prevent subjectivity in the evaluation, we used the option of reproducibility, which is expressed as the ratio of the tracing’s inner area to the respective excursion distance. Tracings with a marked separation between the excursion and incursion, and thus, a greater inner area for the same amount of excursion (10 mm), presented a higher value of reproducibility. In simple words, the higher the value of reproducibility, the lower the quality. Protrusion and mediotrusion showed a statistically significant difference in our study, while open/close movements did not. It is believed that these motions require a higher level of neuromuscular coordination. Another assumption is that protrusive and mediotrusive motions are in close proximity to occlusion relative to open/close movements. Consequently, the probability of exhibiting avoidance mechanisms in these tracings is increased. Kondrat et al. [29] reported reproducibility values of 0.21 and 0.22 in open/close tracings of young, healthy volunteers, which were very close to our corresponding values of 0.24 and 0.25 in the control group. A higher value may be indicative of a greater generalized joint hypermobility [29]. The same hypothesis—that a high separation between the excursion and incursion curves is an early indicator of the hypermobility of the TMJ—was also formulated by Park et al. [35], who examined the bruxing patterns of 50 Japanese subjects and found a higher separation distance in open/close movements and protrusion in those with molar involvement and mediotrusive guidance. The hypermobility of the temporomandibular joint is generally associated with loose ligaments and a lax joint [30]. The other possible reasons for poor quality include damaged or broken electronics, muscle tension, pain, synovial problems (dry joint or increased friction), joint noises (cracking or anything similar, which means a condylar change of pace), and stress during examination [27,30,32,33,34].

A mandibular lateral translation (ΔΥ) can be defined as a translational lateral shift of the mandible. Healthy joints in symmetrical movements such as protrusion and open/close do not typically exhibit such a shift. Therefore, when recorded, it has been proposed that it should be considered an early indicator of intra-articular dysfunction [36]. Fushima et al. [37] investigated the horizontal condylar tracings in the open/close movements of 32 patients diagnosed with disc displacement with reduction, and only two patients presented with tracings that were considered bilaterally normal. Mito et al. [36] examined 168 orthodontic patients who presented with ΔΥ from a sample of 1658 retrospectively selected subjects and 30 subjects to serve as controls. They investigated not only open/close movements, but also mastication and grinding. They concluded that ΔΥ can serve as a diagnostic indicator for the disunion of the proper engagement of the condyle–disk assembly. Greven et al. [38] investigated 56 TMD patients using MRI, condylography, and 12 volunteers and concluded that “The occurrence of transversal, condylar displacement in symmetrical mandibular movements is a strong indicator of temporomandibular disorder (TMD) by the definition of articular “Internal Derangement”. At minimum, a loosening of the capsular and condylar ligaments is existing. The deviation of condylar movement in a quantity of 0.6–0.75 mm indicates limitation of the functional joint space and an internal derangement of the TMJ(s) and is in concordance with the values found in the recent literature”. Therefore, the lateral deviation of the condyle is closely related to internal derangement and may be used as an index for craniomandibular function [35]. Kawagoe et al. [31] examined 240 participants and reported the values from axiographic tracings. Their research was oriented toward the importance of occlusal contact patterns during sleep bruxism. The values for ΔΥ in O/C movements were 0.2 and 0.3 on average, while in P/R, they were 0.2 mm. A total of 72.6% of the participants were free of jaw pain, so the results were rather comparable with those of our control group, which presented similar numbers. Tago et al. [39] examined the transversal deviation during a simulated bruxing movement in 49 patients and reported values of 0.25 to 0.85 mm, with the latter being reported for those with molar involvement, as revealed by Bruxcheckers. Mito et al. [36] reported translation values of 0.18 mm for the control group and 0.72 mm for the “ΔΥ group” for bruxing, and 0.67 and 1.59, respectively, for mastication. We reported values of 0.27 and 0.28 for bruxing and 0.39 (CG) to 0.59 (MG) for mastication.

As presented, our values are in accordance with the values reported in the literature. If one considers 0.6 mm as the cut-off value of mandibular lateral translation for symmetrical movements, then for our study, both the migraineurs and controls fell into this physiological range. However, there was a statistically significant difference in the amount of displacement, with migraineurs presenting higher values for P/R, O/C movements, and mastication. Our cohort was not a cohort of TMD patients. These findings suggest that there is a difference in the functional status of the joint, which might have been compromised in the MG.

The condylar movement velocity provided valuable information about the coordination of movements [17]. In our study, 37% of the MG presented with a two-peak or multi-peak velocity profile for O/C movements. This transient velocity loss, as expressed by the appearance of more than one peak, may be attributed to muscular or articular dysfunction, and if it is constantly present over surveillance, it can be considered a characteristic of TMJ dysfunction [17].

The curvature pattern of the tracings, especially for O/C movements and P/R, is a crucial factor in diagnosing internal derangements of the joints. Normal tracings are concave. Straight or convex tracings are indicative of disc displacement without reduction, while a typical figure-eight tracing is indicative of disc displacement with reduction [40]. In the MG, 6 out of 100 joints for O/C movements and 10 out of 100 for P/R presented a curvature index equal to or less than 0.06. For the CG, the corresponding values were 4/100 and 7/100. However, 30% of the MG presented a “figure-eight” tracing on at least one side, which was indicative of disc displacement with reduction, compared to 10% of the CG; thus, a statistically significant difference was established.

The condylar stability was calculated as the distance from the start point to the end point of the tracing. Since our starting position was the ICP, it can be reasonably assumed that no statistically significant differences would have been established. However, in the MG, there was a greater distance for mediotrusion and protrusion, but not for open/close movements. In simple words, the migraineurs seemed to find it harder to return to ICP after executing more complex movements in terms of neuromuscular coordination. It is not easy to explain this difference; by the end of the tracing, the migraineurs were probably in a position very close to the ICP, and this may have been attributed to muscular dyscoordination, pain, potential occlusal interferences, or even stress and fatigue during the examination.

Every research project is constrained by limitations. This study was not blinded, since every participant possessed knowledge about their assigned group and the objective of the investigation. Additionally, the operator knew to which group each individual belonged. Nevertheless, the jaw-tracking recording process was a series of predetermined steps carried out uniformly for each and every participant, thus limiting the potential influence of this information. Additionally, each step was performed three times during the process. It is worth noting that previous research [25,40] has adequately demonstrated the sensitivity and diagnostic utility of jaw tracking. Jaw tracking is somewhat reliant on the operator, and the interpretation of the results may also be subjective. We circumvented these limitations by relying solely on numerical data and having a trained operator carry out the clinical process on every participant. By doing so, the occurrence of inter-operator error was also prevented.

The main advantages of this study include the use of (a) up-to-date equipment to examine the functionality of the stomatognathic system (Cadiax 4 employs an accurate hinge axis determination and a double-stylus system to digitally depict both the rotational and translational aspects of joint movements) and (b) an experimental group that was representative of migraine patients, since all the participants had been diagnosed and referred by a neurologist. This was also depicted by the 3:1 female-to-male ratio of our groups and by the mean age of the sample, which were achieved unintentionally.

A normal condylographic examination must demonstrate [28,41] (a) a normal quantity; (b) superimposable excursive and incursive tracings; (c) smooth movement with no abrupt changes or velocity disruptions; (d) condylar stability; (e) symmetry; (f) the absence of mandibular lateral translation in symmetrical movements; and (g) superimposable opening, protrusive, and laterotrusive tracings for the first 8 mm of movement. Migraineurs and people who did not suffer from headaches showed statistically significant differences in the functional status of the stomatognathic system in several of the aforementioned criteria.

The notion that painful impulses from the stomatognathic system (including the joint, muscles, and occlusion) are carried through the trigeminal network system into the CNS [4] and may initiate migraine pathophysiology cannot be dismissed. The onset of a migraine attack is often linked to a diverse range of internal and external triggers [42]. It is considered a representation of a modified brain state characterized by heightened excitability, which can activate the trigeminovascular system in individuals with a genetic predisposition. Both migraine pain and craniofacial pain have overlapping pain pathways, with the trigeminovascular system playing a vital role in both. The hypothesis of central sensitization has risen as a means of establishing a correlation between TMDs and migraines. Central sensitization is characterized by an augmented sensitivity of pain-sensing neurons in the central nervous system to their typical or below-threshold incoming signals, as described by the International Association for the Study of Pain “https://www.iasp-pain.org/resources/terminology/” (accessed on 16 December 2023). The subnucleus caudalis (Vc) plays a crucial function in processing both pain sensations from the meninges and noxious stimuli from the orofacial area. The input from the muscles and joints of the jaw adds to the process of central sensitization in the trigeminal nucleus caudalis, which in turn increases the likelihood of experiencing a migraine attack [43]. The relationship between the two should be reciprocal and it is possible that migraine attacks might also decrease the pain threshold in the area of jaw muscles and the joint [44]. An objection could be raised regarding the possibility that such condylographic tracings are caused by the presence of migraines. However, evidence indicates that TMDs and migraines are ailments that often occur together. Therefore, managing and addressing both conditions enhances the treatment outcome [6]. There are reports in the literature [6,45] that the use of orthotic appliances is beneficial for migraine disorders. Our findings support such reports. In the context of our study, statistically significant differences in the mandibular motion analysis between migraineurs and the control group were established. A clinical implication of this finding is that, if migraine patients receive a thorough assessment of their stomatognathic system that identifies symptoms of dysfunction, interventions aimed at addressing these dysfunctions could be advantageous in the concurrent management of their migraines. Future meticulously planned studies on a larger scale, incorporating reversible and/or controlled interventions with the goal of addressing the dysfunction of the stomatognathic system, could be a subsequent step in an effort to further investigate this multifaceted neurological disorder and its possible connection to a dysregulated stomatognathic system.

## 5. Conclusions

The null hypothesis of this study was rejected. The migraineurs presented with statistically significant differences in several aspects of their mandibular motion analysis. The importance of the stomatognathic function in migraine pathogenesis and treatment should be considered by using a patient-centered and interdisciplinary approach.

## Figures and Tables

**Table 1:** Quantity of all tracings, per side and unified. Values are in mm. R: right, L: left, SD: standard deviation, * statistically significant.

Quantity	Migraine Group Mean (SD)	Control Group Mean (SD)	*p*
Protrusion R	9.21 (2.13)	9.84 (2.07)	0.137
Protrusion L	9.65 (2.21)	10.3 (2.01)	0.128
Protrusion R + L	9.43 (2.17)	10.07 (2.04)	0.033 *
Mediotrusion R	10.91 (2.90)	11.53 (2.43)	0.248
Mediotrusion L	11.55 (3.17)	11.99 (2.93)	0.463
Mediotrusion R + L	11.23 (3.04)	11.76 (2.69)	0.241
Open/close R	13.05 (4.59)	13.73 (3.33)	0.394
Open/close L	13.88 (4.18)	14.14 (3.32)	0.729
Open/close R + L	13.46 (4.39)	13.94 (3.32)	0.389

**Table 2:** Quality of all tracings, per side and unified. R: right, L: left, SD: standard deviation, * statistically significant.

Quality (Reproducibility)	Migraine Group Mean (SD)	Control Group Mean (SD)	*p*
Protrusion R	0.25 (0.27)	0.15 (0.12)	0.100
Protrusion L	0.20 (0.19)	0.12 (0.11)	0.001 *
Protrusion R + L	0.22 (0.23)	0.14(0.12)	0.000 *
Mediotrusion R	0.17 (0.13)	0.13 (0.07)	0.183
Mediotrusion L	0.20 (0.15)	0.13 (0.1)	0.003 *
Mediotrusion R + L	0.19 (0.14)	0.13 (0.09)	0.003 *
Open/close R	0.28 (0.19)	0.25 (0.14)	0.953
Open/close L	0.32 (0.29)	0.24 (0.16)	0.450
Open/close R + L	0.30 (0.24)	0.24(0.15)	0.525

**Table 3:** Mandibular lateral translation in symmetrical and functional movements. ΔY was always calculated for medial deviation. Values are in mm. SD: standard deviation, * statistically significant.

Mandibular Lateral Translation (ΔY)	Migraine Group Mean (SD)	Control Group Mean (SD)	*p*
Protrusion	0.38 (0.32)	0.20 (0.21)	0.001 *
Open/close	0.50 (0.34)	0.34 (0.32)	0.031 *
Bruxing	0.27 (0.20)	0.28 (0.18)	0.732
Speech	0.24 (0.24)	0.20 (0.17)	0.690
Mastication	0.59 (0.39)	0.39 (0.22)	0.016 *

**Table 4:** Velocity profiles in protrusion and open/close movements. Two-peak and multi-peak profiles were unified to form one category. * Statistically significant.

Speed Phenomena		Migraine Group	Control Group	*p*
Protrusion	One-peak	56%	72%	0.018 *
	Two-peak	7%	7%	
	Multi-peak	37%	21%	
Open/close	One-peak	63%	83%	0.001 *
	Two-peak	11%	3%	
	Multi-peak	26%	14%	

**Table 5:** Curvature pattern in protrusion and open/close movements and “figure eight” pattern in open/close movements. Values of less than 0.05 represent a straight tracing while higher values indicate a curved tracing. * Statistically significant.

Curvature Pattern	Migraine Group Mean (SD)	Control Group Mean (SD)	*p*
Protrusion R Kob’s coefficient	0.08 (0.04)	0.09 (0.03)	0.177
Protrusion L Kob’s coefficient	0.09 (0.04)	0.09 (0.03)	0.975
Open/close R Kob’s coefficient	0.17 (0.06)	0.17 (0.05)	0.686
Open/close L Kob’s coefficient	0.17 (0.06)	0.17 (0.06)	0.810
Figure 8 in Open/close n (%)	15 (30%)	5 (10%)	0.012 *

**Table 6:** Condylar stability of all tracings, per side and unified. Condylar stability was calculated as distance between start point and end point of tracing. Values are presented in mm. R: right, L: left, SD: standard deviation, * statistically significant.

Condylar Stability	Migraine Group Mean (SD)	Control Group Mean (SD)	*p*
Protrusion R	0.24 (0.16)	0.19 (0.15)	0.066
Protrusion L	0.26 (0.18)	0.17 (0.12)	0.004 *
Protrusion R + L	0.25 (0.17)	0.18 (0.13)	0.001 *
Mediotrusion R	0.37 (0.52)	0.19 (0.15)	0.020 *
Mediotrusion L	0.40 (0.49)	0.22 (0.16)	0.065
Mediotrusion R + L	0.39 (0.50)	0.21 (0.15)	0.003 *
Open/close R	0.33 (0.30)	0.25 (0.16)	0.458
Open/close L	0.34 (0.39)	0.26 (0.21)	0.801
Open/close R + L	0.33 (0.35)	0.25 (0.19)	0.422

## Data Availability

The data presented in this study are available from the corresponding author upon request due to privacy and ethical reasons.
